# Febuxostat Enhances the Antitumor Efficacy of 2-Fluoroadenine and 5′-Methylthioadenosine in *MTAP*-Deleted Cancer

**DOI:** 10.1158/2767-9764.CRC-26-0193

**Published:** 2026-08-14

**Authors:** Baiqing Tang, Hyung-Ok Lee, Daniel Krzizike, Kathy Q. Cai, Sapna Gupta, Warren D. Kruger

**Affiliations:** 1 Cancer Signaling and Microenvironment Program, Fox Chase Cancer Center, Philadelphia, Pennsylvania.; 2 Cancer Prevention and Control Program, Fox Chase Cancer Center, Philadelphia, Pennsylvania.

## Abstract

Homozygous deletion of the methylthioadenosine phosphorylase (*MTAP*) gene is a frequent genetic alteration in cancer. The MTAP enzyme, which creates adenine from 5′-methylthioadenosine (MTA), is constitutively expressed in all tissues throughout the body. Previously, we described a novel strategy to specifically target *MTAP*-deleted cancer cells by combining the antipurine prodrug 2-fluoroadenine (2FA) with MTA. *In vitro*, this combination efficiently kills *MTAP*− cancer cells while leaving *MTAP*+ cells unharmed. However, *in vivo*, the combination was less effective at tumor cell killing, resulting in slower growth but not tumor regression. In this study, we show that this effectiveness difference is associated with a large difference in the amount of 2FA that is incorporated into 2FA-containing nucleotides. To explain this observation, we tested the hypothesis that 2FA was a target for the purine metabolic enzyme xanthine oxidase (XO), which is found in various extracellular fluids. *In vitro*, the addition of XO protein to culture media effectively abolishes killing by 2FA, and this effect can be fully reversed by the addition of febuxostat (FX), a XO inhibitor. *In vivo*, the addition of FX to 2FA results in increased cell killing and toxicity, and a 1,000% increase in the amount of 2FA that is converted to its active form, 2FA-monophosphate. Xenograft studies using *MTAP*− HT1080 and MiaPaCa-2 cell lines show that a 2FA/MTA/FX cocktail can cause tumor regression *in vivo*. These studies suggest that a combination of 2FA/MTA/FX may be useful in treating *MTAP*− cancer.

**Significance:**

Deletion of the *MTAP* gene is one of the most frequent genetic alterations in cancer, making it a potentially good target for cancer therapy. Previously, we demonstrated that a combination of 2FA and MTA could specifically target *MTAP*− cells. In this study, we show that the addition of FX substantially increases the potency of 2FA/MTA *in vivo*.

## Introduction

Methylthioadenosine phosphorylase (MTAP) is a key enzyme in the purine and methionine salvage pathways that converts the polyamine byproduct 5′-methylthioadenosine (MTA) into adenine and methylthioribose-1-phosphate. It is a so-called housekeeping enzyme, expressed constitutively in all tissues (1). The gene encoding human *MTAP* is located on chromosome 9p21, about 80 kb away from the *CDKN2A/ARF* region (2, 3). Importantly, homozygous deletion of *MTAP* is one of the most frequent genetic alterations observed in cancer, with about 12% of all tumors exhibiting *MTAP* loss (4). Homozygous *MTAP* loss is especially frequent in some of the most lethal cancers, including brain, pancreas, esophageal, and lung (5). The high frequency of *MTAP* loss in tumor cells has led to much interest in trying to develop novel therapeutic strategies that specifically target *MTAP* deletion in cells (6).

**Figure 1.**
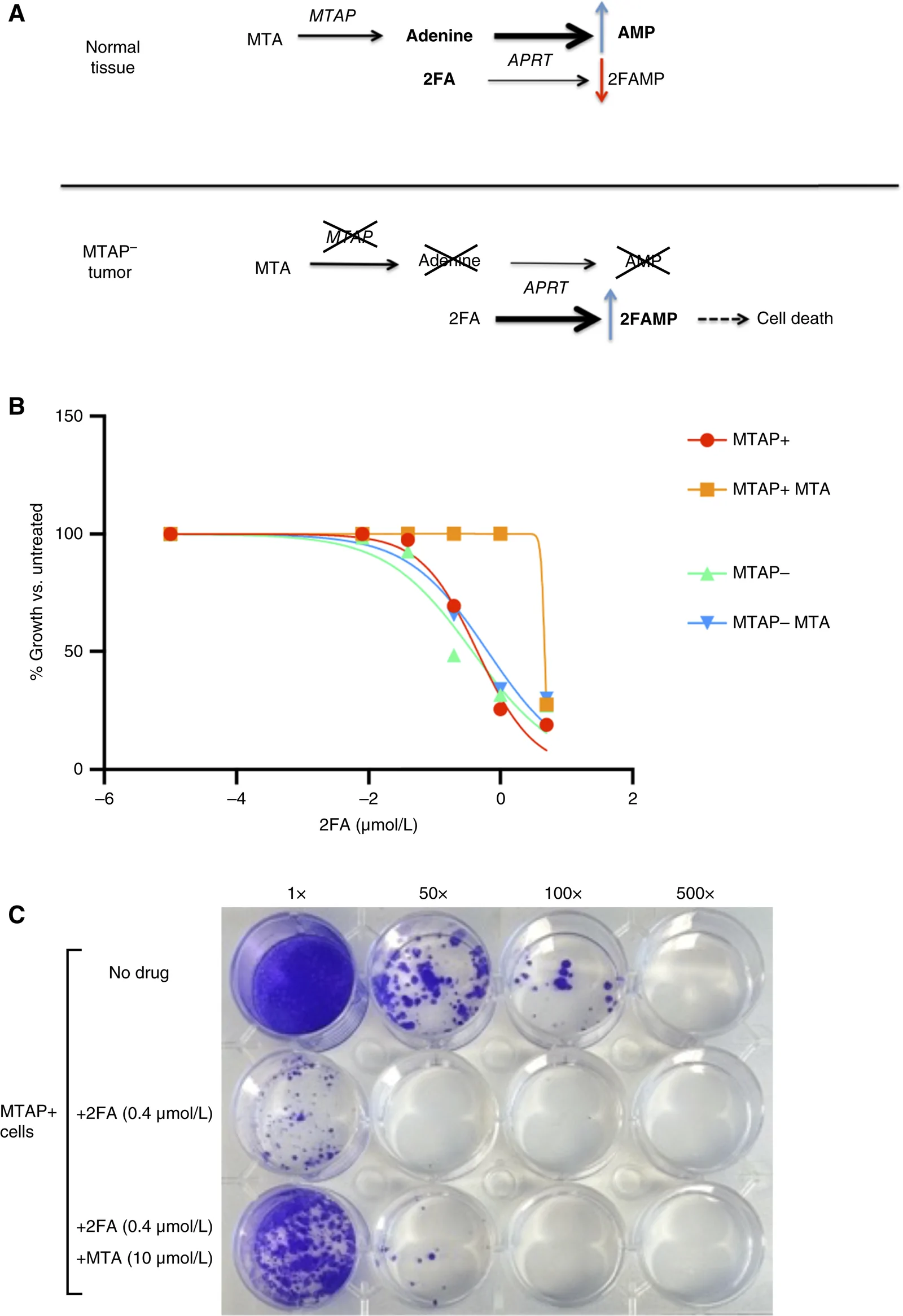
*MTAP*-mediated chemo-protection strategy. **A,** Scheme showing interaction between MTA and 2FA metabolism in *MTAP*+ and *MTAP*− cells. **B,** MTA protection in isogenic *MTAP*+ and *MTAP*− immortalized human pancreatic epithelial (HPNE) cells treated with 2FA in the presence or absence of 10 μmol/L MTA for 48 hours. **C,** Colony formation showing MTA protection in mouse-derived KPC3-3 pancreatic cancer (*MTAP*+) cells.

Previous work from our lab has explored the use of a two-drug combination to specifically target *MTAP*-deleted cells (7). In this approach, a potent nonspecific cell-killing base analogue, 2-fluoroadenine (2FA), is combined with the MTAP substrate MTA, which acts as a protecting agent for *MTAP*+ cells, but not for *MTAP*− tumor cells. We refer to this approach as the “MTAP-mediated chemo-protection strategy” (Fig. 1A). In *MTAP*+ normal tissue, MTA is cleaved to adenine, which in turn is converted to adenine monophosphate (AMP) by the action of the enzyme adenine phosphoribosyltransferase (APRT). 2FA is an analogue of adenine that is a prodrug, which must be converted into 2FA-monophosphate (2FAMP) by APRT to become cytotoxic. Once 2FAMP is formed, it can be further converted to 2FA-disphosphate (2FADP) and 2FA-triphosphate (2FATP; ref. 8). When *MTAP*+ cells are incubated with a five or more molar excess of MTA to 2FA, the adenine produced from MTA competes with 2FA for APRT binding, resulting in reduced production of 2FAMP. In contrast, in *MTAP*− tumor cells, MTA is not converted to adenine, resulting in no competition for APRT and a greater percentage of the 2FA being converted to 2FAMP. In cell culture, the 2FA/MTA combination kills *MTAP*− cells very efficiently after 48 hours but is much less toxic to *MTAP*+ cells (Fig. 1B and C). However, in mice, we found that the effectiveness of 2FA in killing tumor cells was much reduced, resulting in only a modest difference in tumor growth kinetics between *MTAP*+ and *MTAP*− xenografts in 2FA/MTA-treated mice (7).

In the current study, we explore the hypothesis that the reduced effectiveness of 2FA in tumor cell killing *in vivo* may be due to the presence of the purine metabolic enzyme xanthine oxidase (XO). We show that 2FA toxicity is modulated by XO and that the addition of the FDA-approved XO inhibitor febuxostat (FX) greatly enhances the ability of 2FA/MTA to regress tumor growth *in vivo* in two different mouse xenograft models.

## Materials and Methods

### Reagents

For cell culture studies, 2FA (Oakwood Chemical) and MTA (Sigma-Aldrich) were dissolved in DMSO and then diluted in cell culture medium. 2FA was dissolved at a concentration of 0.36 mg/mL in the presence of equal normality sulfuric acid in a 1% carboxymethylcellulose solution. MTA was added to the 2FA solution at a concentration of 1.8 mg/mL. XO (Sigma-Aldrich) was obtained as a lyophilized powder and used at a concentration of 5 U/L (U = μmole xanthine oxidized/minute). FX was obtained from Sigma-Aldrich.

### Cell line studies

Isogenic HT1080 *MTAP*+/*MTAP*− cells were created and maintained as described (9). KPC3-3 cells (*MTAP*+) were derived as described (10). MiaPaCa-2 cells were obtained from ATCC. All cell lines were validated by short tandem repeat analysis and tested for mycoplasma using the PCR Mycoplasma Detection Kit (Thermo Fisher Scientific). Isogenic *MTAP*+/*MTAP*− HPNE cells (11) were created using CRISPR knockout as described (7). All cell lines had MTAP status confirmed by Western blot. Foci formation assays were performed as previously described (12). Drug sensitivity studies were done using MTT assays 48 hours after drug exposure as described previously (7).

### LC/MS-MS adenine and fluoroadenine nucleotide quantitation

**Figure 2.**
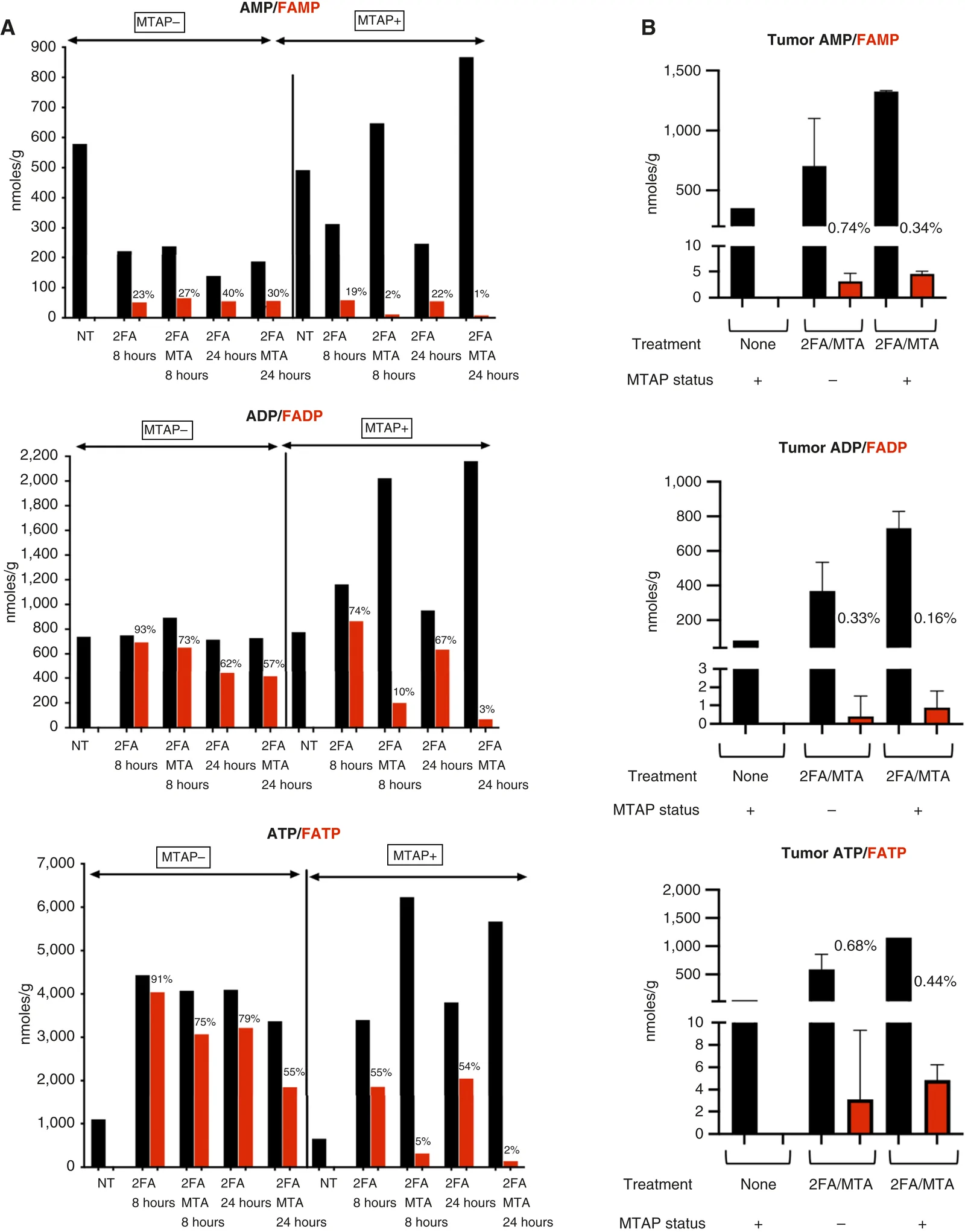
Nucleotide concentrations. **A,** Mono-, di-, and trinucleotide levels in isogenic *MTAP*+ and *MTAP*− HT1080 cells treated with nothing (NT), 2 μmol/L 2FA alone, or with 10 μmol/L MTA. Length of time of treatment was either 8 or 24 hours. Black bars show adenine nucleotides, whereas red bars show 2FA nucleotides. The percentage of 2FA relative to adenine for each nucleotide is shown above the red bars. **B,** Nucleotide levels from HT1080 *MTAP*+ and *MTAP*− tumors (*n* = 3) grown in SCID mice treated with either 2FA alone or 2FA + MTA. Error bars show SEM.

Cell pellets were lysed in 500 μL of 12% perchloric acid, which was spiked with 10 nmol of ^15^N_5_-AMP (Sigma), followed by centrifugation to precipitate proteins. Tumor tissues (Fig. 2) on the other hand were homogenized in 12% perchloric acid spiked with an internal standard to make a 10% homogenate, followed by centrifugation to precipitate protein. The supernatant was further neutralized with KOH to remove perchlorate by centrifugation, diluted to 2 mL with H_2_O, and then subjected to solid phase extraction using a 1 cc Oasis WAX cartridge (Waters). Solid phase extraction cartridges were conditioned with methanol and then equilibrated with 50 mmol/L ammonium acetate (pH 4.5). After loading the samples, the cartridges were washed with ammonium acetate and eluted with 0.8 mL of methanol/water/ammonium hydroxide (80:15:5). Elution was then dried in SpeedVac and resuspended in a 50:50 water/acetonitrile solution. After drying and reconstitution, samples were analyzed by LC/MS-MS using a Waters ACQUITY I-Class UPLC coupled to a Waters Xevo TQ-S micro tandem quadrupole mass spectrometer. Chromatographic separation was achieved using a Waters ACQUITY BEH Amide column (2.1 × 150 mm, 1.7 μm) at a flow rate of 0.2 mL/minute using a linear gradient from 80% to 70% mobile phase A over 12 minutes at 45°C. Mobile phase A consisted of 15 mmol/L ammonium acetate and 0.3% ammonia in 90% acetonitrile, and mobile phase B consisted of 15 mmol/L ammonium acetate and 0.3% ammonia in water (all solvents were Optima LC/MS grade, Fisher Scientific). For the MS-MS detection, analytes were monitored using negative mode electrospray ionization with the following MRM transitions: AMP, m/z 346.03 → 78.87; ADP, m/z 425.97 → 133.92; ATP, m/z 505.97 → 158.83; 2FAMP, m/z 364.03 → 131.92; 2FADP, m/z 443.97 → 151.92; 2FATP, m/z 523.97 → 158.83; and N^15^-AMP, m/z 351.01 → 78.87. LC/MS-MS data were processed using Waters MassLynx 4.2. Control experiments examining the linearity and sensitivity of the assay found that the assay was linear (R^2^ > 0.98) for all the metabolites tested between 2 and 2,000 pmoles. The solid phase extraction step was omitted for the mouse tissue experiments shown in Fig. 5, and therefore, only the monophosphates (2FAMP and AMP) could be quantitated.

### Mouse studies

C57BL6 and SCID (C.B-*Igh-1*^*b*^/IcrTac-*Prkdc*^*scid*^) were obtained from Taconic Biosciences, whereas nude (NU/J) mice were obtained from The Jackson Laboratory. All studies were reviewed and approved in compliance with the Fox Chase Cancer Center Institutional Animal Care and Use Committee (IACUC), protocol #22-12. Mice were given drugs by oral gavage at concentrations and timing indicated in the figure legends. At the end of the experiments, mice were euthanized, and tissues were collected, fixed in buffered formalin, and embedded in paraffin. The paraffin blocks were cut into 5-μm-thick sections that were placed on positively charged slides. Sections were stained with hematoxylin and eosin and mounted with Permount (Fisher Scientific). For caspase 3, Ki-67, and myeloperoxidase, IHC staining was done as described previously (7).

Either SCID or nude mice were injected in the hind flank with 2 × 10^7^ tumor cells in 200 μL of Matrigel. After 7 days, tumor size was measured with calipers (13), and mice were randomly divided into vehicle and treatment groups. Mice were then treated with either vehicle or drug combination according to the schedule indicated in the figures. Tumor growth was calculated by comparing the difference in volume at T_n_ with the volume at the time of the start of treatment (T_0_).

Mice were euthanized by isoflurane overdose followed by cervical dislocation in accordance with IACUC guidelines.

### Statistics

Pairwise comparisons between groups were performed using a Student two-sided *t* test. A *P* value of 0.05 or less was considered significant. For tumor growth experiments, a single-sided ANOVA was used followed by Dunnett *post hoc* test against vehicle-treated mice at each time point. All graphs show standard error of the mean (SEM). Different letters on points of graphs indicate significant differences in the *post hoc* test. The number of replicates used for each experiment is shown in the appropriate figure legend.

## Results

### 2FA incorporation in adenine nucleotides *in vitro* versus *in vivo*

To understand why 2FA/MTA was so much more effective in killing *MTAP*− tumor cells *in vitro* versus *in vivo*, we measured the ratio of 2FA-containing adenine nucleotide pools (2FANP) to adenine nucleotide pools (ANP) in lysates from isogenic *MTAP*+ and *MTAP*− HT1080 cells treated with either 2 μmol/L 2FA alone or 2FA + 10 μmol/L MTA (Fig. 2A). In both *MTAP*+ and *MTAP*− cells, 2FA treatment resulted in significant production of 2FA-containing nucleotides (2FANP), with their overall concentration being between 30% and 70% of the respective endogenous ANPs at 24 hours. When a 5-fold molar excess of MTA was added, we reduced the 2FANP/ANP ratio by more than 5-fold in *MTAP*+ cells but saw no effect in *MTAP*− cells. These findings support the hypothesis that MTA inhibits the conversion of 2FA to 2FANP and that an increase in the 2FANP/ANP ratio causes increased cell killing.

We next examined extracts derived from subcutaneous xenograft tumors from the same cell lines in mice treated with either nothing or given four doses of 2FA/MTA (10 mg/kg 2FA, 50 mg/kg MTA) over an 8-day period (7). In the tumors, we found that the concentration of 2FNP versus ANP was less than 0.8% (Fig. 2B) in both *MTAP*+ and *MTAP*− tumors. Importantly, the amount of 2FA given to the mice is 10 times higher than that used in the cell culture experiment (making the highly conservative assumption that a mouse is 100% water). Taken together, these findings suggest that 2FA incorporation in nucleotide pools is far less efficient *in vivo* than *in vitro*.

### XO inhibits cell killing by 2FA

**Figure 3.**
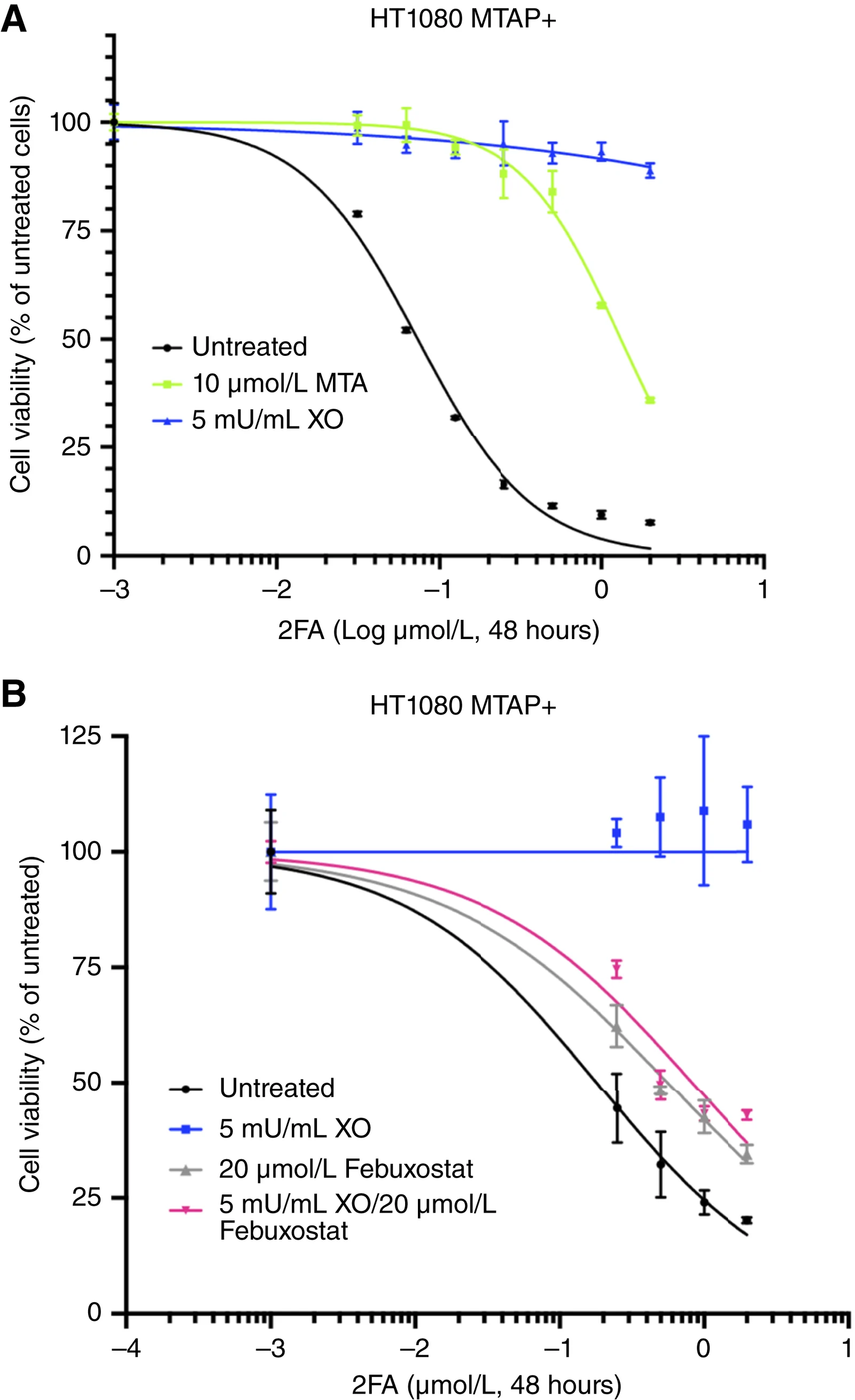
XO inhibits 2FA toxicity. **A,** Growth inhibition of HT1080 *MTAP*+ cells treated with various concentrations of 2FA for 48 hours in the presence or absence of either MTA or XO. **B,** Effect of the XO inhibitor FX on XO protection. *N* = 4 per point; error bars show SEM. Different letters next to points indicate significant differences.

Based on the above findings, we hypothesized that there may be specific factors *in vivo* that could reduce 2FA bioavailability and thereby explain the low amounts of 2FANP concentrations and the limited effectiveness of 2FA/MTA to inhibit *in vivo* tumor growth. XO is a purine catabolic enzyme that catalyzes the oxidation of hypoxanthine and xanthine to uric acid. Clinically, XO inhibitors such as allopurinol or FX are used to treat gout (14). In humans and mice, XO is found at high levels in the intestine and liver but is also found at significant levels in extracellular fluids, such as serum (15). XO is either not expressed or expressed at very low levels in tumor-derived cell lines (16). Because XO is known to have relatively low substrate specificity and can oxidize adenine to form 2,8-dihydroxyadenine (17), we tested whether XO addition could protect cells against 2FA-mediated cell killing. Addition of XO to media effectively protected HT1080 (*MTAP*+), MiaPaCa-2 (*MTAP*−), and AsPC-1 (*MTAP*−) from 2FA killing *in vitro*. This protection was entirely reversed by adding the XO inhibitor FX (Fig. 3; Supplementary Fig. S1). As expected, the addition of FX had minimal effect on the growth of cells by themselves (Supplementary Fig. S2). These results demonstrate that XO can efficiently protect cells from 2FA.

### Effects of FX on 2FA toxicity in mice

To determine whether inhibition of XO could enhance the *in vivo* activity of 2FA, we evaluated the combined toxicity of 2FA and FX in mice. Animals were treated daily by oral gavage with 40 mg/kg 2FA, 5 mg/kg FX, or the combination. Body weight and clinical appearance were monitored throughout the experiment.

**Figure 4.**
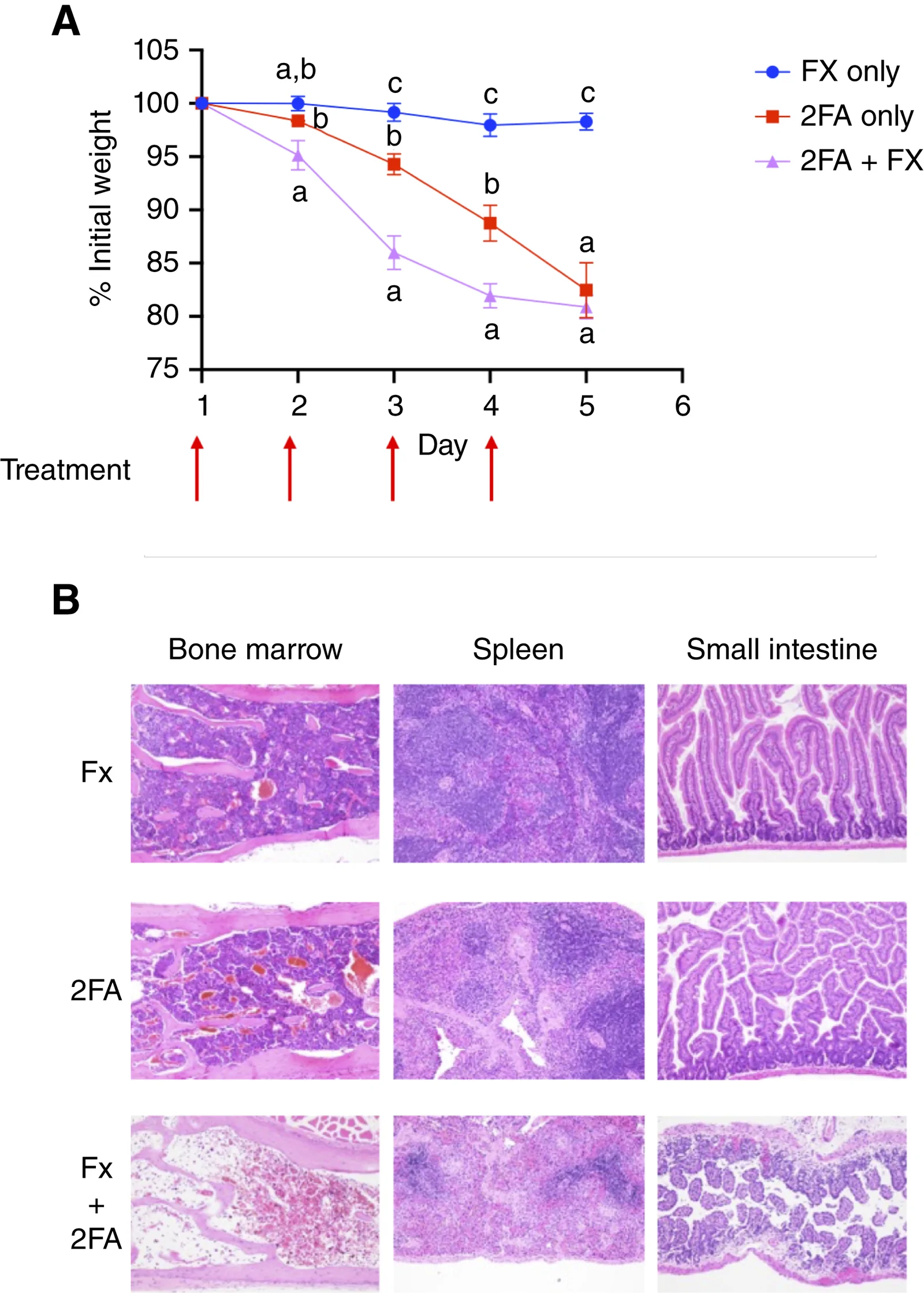
FX increases 2FA tissue toxicity in mice. Male 15-week-old C57BL6 mice were given orally either 40 mg/kg 2FA alone, 5 mg/kg FX alone, or both (*n* = 4). Up arrows indicate treatment days. **A,** Percent body weight change in mice treated with the indicated drugs. Different letters next to points indicate significant differences. **B,** Representative images of the spleen, bone marrow, and small intestine of different groups (160× magnification).

By day 4, mice receiving the combination treatment exhibited marked lethargy and greater weight loss than those treated with 2FA or FX alone. Due to these observations, the dose of both agents was reduced by 25% in the combination group. Despite this adjustment, two mice in the 2FA/FX cohort were found deceased on the morning of day 5 (Supplementary Video S1), prompting early termination of the study. Consistent with these clinical findings, animals treated with the drug combination showed significantly greater weight loss compared with the single-agent group (Fig. 4A). Histopathologic analysis further demonstrated increased tissue toxicity associated with combined treatment (Fig. 4B). Examination of bone marrow, spleen, and colon revealed pronounced cellular depletion and architectural disruption in 2FA/FX-treated mice, whereas tissues from animals receiving 2FA or FX alone showed only mild or no abnormalities.

**Figure 5.**
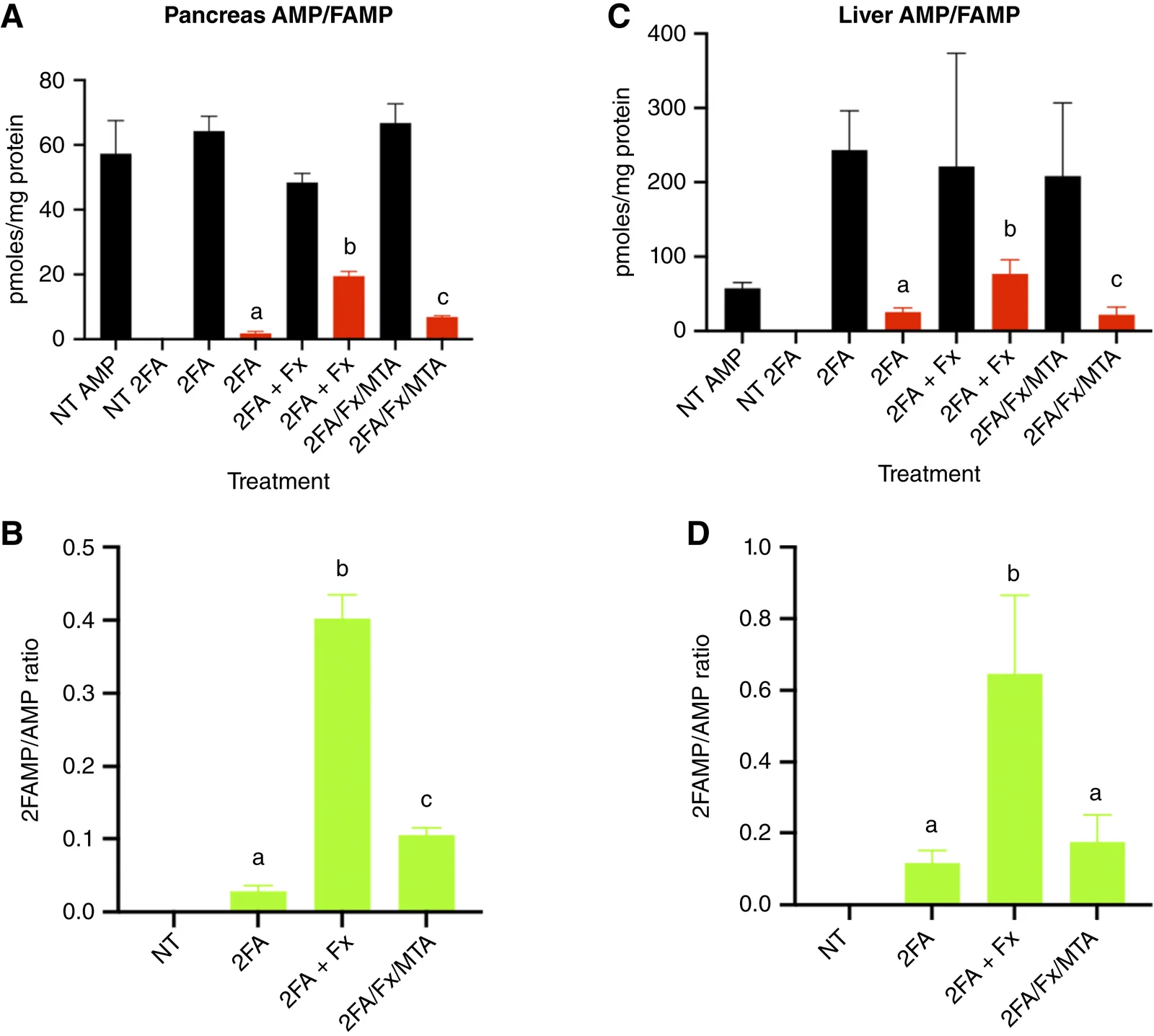
Tissue 2FAMP concentrations. **A,** Concentration of AMP and 2FAMP in pancreas tissue from mice given 2FA (30 mg/kg), 2FA + FX (3.75 mg/kg), 2FA + FX + MTA (180 mg/kg), or nothing (NT). Mice were treated once per day for 2 days, and tissue was harvested on day 3. *n* = 3 per group; error bars show SEM. **B,** 2FAMP/AMP ratio for each group. **C,** Concentration of AMP and 2FAMP in liver. **D,** Ratio in liver. Different letters next to points indicate significant differences.

We also examined the effect of FX on 2FAMP formation *in vivo* in a separate experiment. Mice were treated with vehicle, 2FA, 2FA/FX, or 2FA/MTA/FX for 2 consecutive days, and the pancreas and liver were harvested on the third day. Tissue was then assessed for 2FAMP and AMP concentrations (Fig. 5). The addition of FX to 2FA resulted in a 1,000% increase in the 2FAMP/AMP ratio in both the pancreas and liver. Upon MTA addition, these ratios decreased by 75%. These studies show that FX enhances and MTA suppresses the conversion of 2FA to 2FAMP.

Together, these findings indicate that FX markedly enhances the systemic toxicity of 2FA *in vivo*. This increased toxicity is consistent with the hypothesis that XO normally limits 2FA bioavailability and that its inhibition results in higher levels of active fluoroadenine metabolites in normal tissues.

### 2FA/FX/MTA inhibits xenograft tumor growth in immunocompromised mice

**Figure 6.**
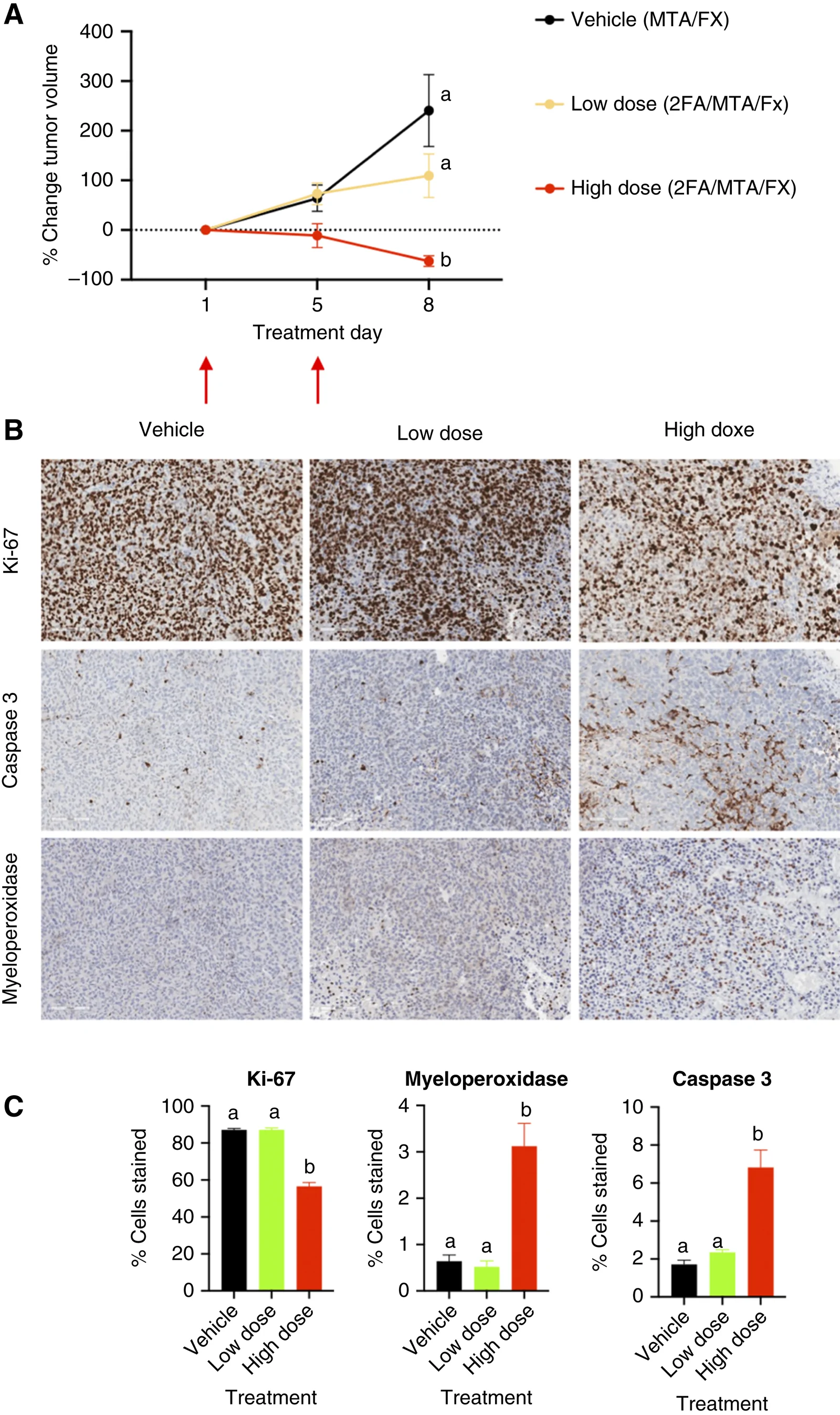
Growth of HT1080 cells in SCID mice. **A,** Average tumor volume in vehicle (no 2FA, 180 mg/kg MTA, 3.75 mg/kg FX), low-dose treatment (10 mg/kg 2FA, 90 mg/kg MTA, 3.75 mg/kg FX), and high-dose treatment (20 mg/kg 2FA, 180 mg/kg MTA). Arrows show the days when the drug was administered. *n* = 4 per group. Note: One vehicle-treated mouse was found dead on the day before the end of the study. **B,** Representative images of tumors stained for Ki-67, myeloperoxidase, and caspase 3. **C,** Quantitation of staining. Six to eight fields were quantitated for each sample.

We next tested if 2FA/MTA/FX improves tumor control in *MTAP*-deleted xenografts. First, we tested for tumor growth inhibition using HT1080 *MTAP*− fibrosarcoma cells implanted in SCID mice. This model was identical to that used in our previous study (7), allowing us to see if the addition of FX increased the effectiveness of the 2FA/MTA treatment. Tumor cells were injected into the flank of 12 mice, and tumor size was determined after 8 days. Mice were then divided into three groups (*n* = 4) and treated with either vehicle (MTA/FX, 180/3.75 mg/kg), low-dose 2FA/MTA/FX (10/90/3.75 mg/kg), or high-dose 2FA/MTA/FX (20/180/3.75 mg/kg). A second dose was administered 4 days later, and on the eighth day, mice were euthanized and tissue was collected. Tumors in the high-dose group exhibited a 60% shrinkage in tumor size, whereas tumors in the vehicle group increased by 220% (Fig. 6A). The low-dose treatment group tumors were slightly smaller than the vehicle-treated tumors, but this was not statistically significant. Tumors treated with high-dose 2FA/MTA/FX showed decreased levels of the proliferation marker Ki-67 and increased levels of the apoptosis marker caspase 3 and the inflammation marker myeloperoxidase (Fig. 6B and C). These findings indicate that high-dose 2FA/MTA/FX can shrink HT1080 xenograft tumors, and not simply slow growth. Toxicity of the treatment was assessed by monitoring the animals’ weight and histopathologic examination of the thymus, spleen, intestine, and bone marrow at the end of the experiment. We observed increased weight loss and increased tissue toxicity in the 2FA-treated compared with the vehicle-treated animals (Supplementary Fig. S3). This toxicity was more evident in the high-dose cohort.

**Figure 7.**
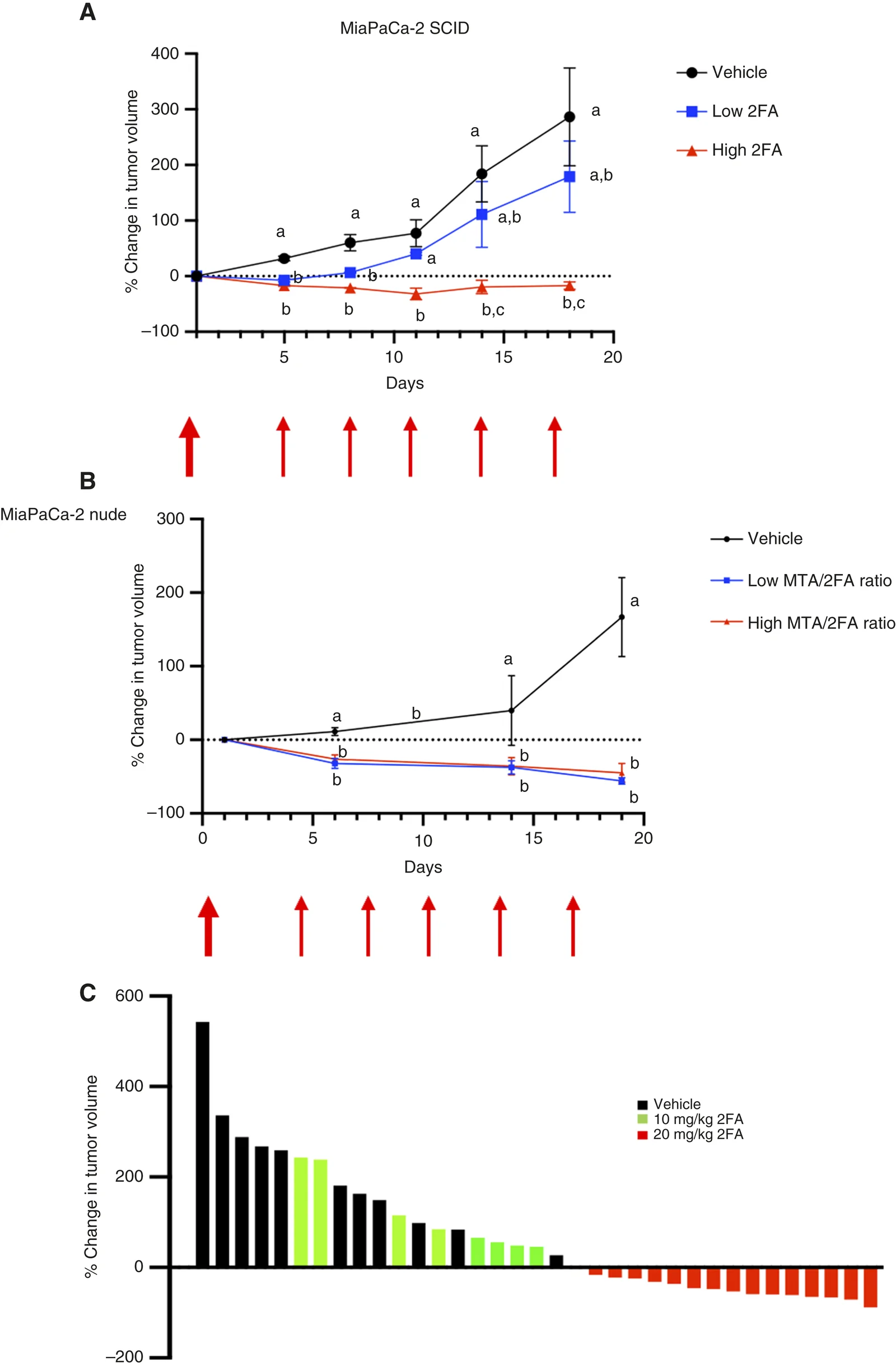
Growth of MiaPaCa-2 cells in mice. **A,** Tumor growth in SCID mice treated with either a high 2FA mix (20 mg/kg 2FA, 180 mg/kg MTA, 3.75 mg/kg FX), a low 2FA mix (10 mg/kg 2FA, 180 mg/kg MTA, 3.75 mg/kg FX), or vehicle (180 mg/kg MTA, 3.75 mg/kg FX). Arrows show the time of treatment. After the initial dose, concentrations of 2FA were halved for both the high- and low-dose groups. **B,** Tumor growth in nude mice treated with either a low 2FA/MTA ratio (20 mg/kg 2FA, 180 mg/kg MTA, 3.75 mg/kg FX), a high 2FA/MTA ratio (20 mg/kg 2FA, 360 mg/kg MTA, 3.75 mg/kg FX), or vehicle (180 mg/kg MTA, 3.75 mg/kg FX). Again, 2FA concentrations were halved after the first dose. **C,** Waterfall plot for all xenografts combined.

The second and third experiments examined the effectiveness of 2FA/MTA/FX in treating tumors formed by MiaPaCa-2 cells, an *MTAP*− pancreatic adenocarcinoma–derived line. Cells were implanted in 12 SCID mice and allowed to grow for 1 week. Unfortunately, during this week, one mouse had to be euthanized due to fight wounds. Mice were then treated with either vehicle (MTA/FX, 180/3.75 mg/kg) or two different doses of the 2FA/MTA/FX cocktail, varying in the amount of 2FA but maintaining the 2FA/MTA ratio (10/180/3.75 mg/kg or 20/360/3.75 mg/kg). After the first dose, it seemed that the high-dose animals were suffering some toxicity, so for all subsequent doses, the 2FA concentration was halved. Tumors treated with high-dose 2FA/MTA/FX decreased in size during the treatment, and by the end of the treatment, they were less than half the size of the tumors in untreated and low-dose mice (Fig. 7A; Supplementary Fig. S4A). During the treatment, two animals (one in the high-dose group and one in the low-dose group) were judged morbid and euthanized before the end of the experiment. Both animals exhibited pronounced weight loss after the start of treatment (Supplementary Fig. S4B). However, the other mice in both treatment groups showed either no or minimal weight loss, so it was not clear whether these deaths were treatment-related, tumor-related, or due to some other cause. H and E tissue analysis of the colon, liver, kidney, thymus, spleen, and bone marrow was performed on vehicle and high-dose mice at the end of the study. No significant differences were observed for any of the tissues, apart from the bone marrow, in which the one high-dose mouse that was euthanized early showed significant levels of toxicity (Supplementary Fig. S4C).

Because of the loss of three animals during the experiment, we performed a second study using the same cell line but with nude mice instead of SCID mice. Mice were treated with either vehicle (3.75 mg/kg FX, 180 mg/kg MTA) or two different ratios of 2FA/MTA (20/180 or 20/360 mg/kg). Like the first experiment, we reduced the amount of 2FA by 50% after the first dose. Tumors in both treatment arms were significantly different from the vehicle arm, showing shrinkage compared with vehicle-treated animals (Fig. 7B; Supplementary Fig. S5A and S5B). However, reducing the 2FA/MTA ratio (i.e., increasing the amount of MTA) did not seem to offer a benefit with regard to weight loss (Supplementary Fig. S5C).

Taking the three different treatment experiments together, we found that all the mice that were treated with at least one dose of 20 mg/kg 2FA FX had tumor shrinkage during the experiment (Fig. 7C). The mean amount of tumor reduction in tumor volume in these animals was 47%, with 13 out of 17 showing a 30% or greater reduction.

## Discussion

Homozygous deletion of the *MTAP* gene is one of the most frequent genetic rearrangements observed in cancer, with especially high rates in several tumor types with very low 5-year survival rates, including glioblastoma (loss rate 26%–60%), pancreatic ductal adenocarcinoma (18%–64%), cholangiocarcinoma (15%–30%), esophageal cancer (12%–27%), and non–small cell lung cancer (12%–38%; ref. 5). Therefore, targeting this alteration for cancer treatment is an area of intense interest. In our previous work, we developed a strategy in which we combined a “killing agent” (2FA) and an MTAP-requiring protecting agent (MTA) to specifically target *MTAP*-deleted tumor cells (Fig. 1A). In cell culture models, the 2FA/MTA combination was highly effective, but *in vivo*, it was less so, only slowing *MTAP*− tumor growth and not actually reducing tumor size (7). Here, we showed that this difference in behavior was associated with a large difference in the amount of 2FA that gets converted to 2FAMP in the two different systems. *In vitro*, the 2FAMP/AMP ratio is between 0.2 and 0.4, whereas *in vivo* the ratio is only about 0.0074, representing at least a 30-fold reduction. Given this finding, we hypothesized that 2FA was being metabolized *in vivo* before being taken up by the tumor cell. A likely candidate enzyme for this process is XO, which can oxidize several different purines, including xanthine, hypoxanthine, and adenine (18). XO is also known to be present at detectable levels in mouse serum (15). Consistent with this idea, we found that the addition of the XO enzyme could entirely reverse 2FA-induced cell killing *in vitro* and that this effect could be reversed by the simultaneous addition of the specific XO inhibitor FX. To test the idea that XO in serum or the intestine might be inactivating 2FA *in vivo*, we combined 2FA with FX and examined toxicity in mice. We observed that FX addition substantially increased 2FA toxicity and resulted in significantly more 2FAMP being produced *in vivo*. In addition, we demonstrated that when MTA was added to a mixture, 2FAMP levels were reduced. Our findings show that 2FA toxicity *in vivo* can be increased by FX and reduced by MTA.

We next performed a series of experiments examining the effects of various concentrations of the 2FA/MTA/FX combination on three different *MTAP*− cancer xenograft models. Seventy-six percent (13/17) of mice treated with at least one dose of 20 mg/kg 2FA in combination with MTA showed at least a 30% decrease in tumor volume. However, we did not see tumor regression using lower (10 mg/kg) doses of 2FA, suggesting that the treatment is dose-dependent. Overall, in comparison with our earlier studies using only 2FA/MTA, we find that the addition of FX significantly enhances the antitumor activity.

However, we also found that 2FA/MTA/FX was more toxic than 2FA/MTA. In all three of the treatment studies, treated mice had weight loss and some atrophy in the bone marrow and spleen compared with vehicle-treated mice. This was somewhat expected, as the mechanism of MTA protection in *MTAP*+ cells essentially involves competition between adenine and 2FA for conversion to their respective monophosphates by the APRT enzyme. Mice treated with only 2FA/MTA would have a significantly lower 2FA/adenine ratio compared with mice treated with 2FA/MTA/FX. Thus, to achieve the same level of MTA protection, we would have had to increase the amount of MTA. Due to solubility constraints, we were limited to a maximum dose of 360 mg/kg. Future studies could examine improved formulations of MTA or perhaps a dosing schedule in which MTA is given alone between doses of 2FA.

Although we did lose four mice during the treatment stage, two of the four mice were vehicle-treated, suggesting that this was not due to 2FX toxicity. In our experience, immunocompromised animals implanted with tumors do sometimes die unexpectedly, but we cannot rule out the possibility that these mice might have had a reaction to MTA, FX, or perhaps the combination. However, neither MTA nor FX has been shown to have significant toxicity in rodents near the concentration ranges used here (19–21). With regard to the optimal ratio of 2FA/MTA, we did not observe any difference in effectiveness between a 1/3.5 and a 1/7 mol/L ratio of 2FA/MTA, but the numbers were very small (*n* = 4/group), and this needs to be explored further. It is also important to point out that in this study, the cell lines we tested, HT1080 and MiaPaca-2, are both *MTAP*−; thus, we cannot be certain that the tumor regression we observed is specific to *MTAP*− cells. However, in our earlier studies (7), in which we tested 2FA + MTA alone, we observed a strong effect on the growth inhibition of tumors by MTAP status.

Another approach to target *MTAP* loss involves the use of drugs that inhibit protein symmetric dimethyl arginine methylation by targeting PRMT5, the methyltransferase that carries out this reaction. Second-generation PRMT5 inhibitors have been developed that cooperatively bind with MTA to the PRMT5 active site and have shown promise in inhibiting the growth of *MTAP*− tumor cells in mouse models and in early human clinical trials. Recently, it has been demonstrated in an orthotopic mouse model of pancreatic cancer that combining a PRMT5 inhibitor with the DNA-damaging agent gemcitabine (2′, 2′-difluoro-2′-deoxycytidine) produced more tumor growth inhibition than gemcitabine alone (22). Given that gemcitabine and 2FA are both nucleoside analogs, it might be interesting to add a PRMT5 inhibitor to the 2FA/MTA/FX combination. Because both treatments have increased specificity for *MTAP*− cells, the combination may have an even greater therapeutic window.

In summary, we show here that the addition of FX to 2FA/MTA enhances tumor cell killing *in vivo*. Given the high frequency of *MTAP* loss in a variety of tumor types, we believe this combination should be explored further.

## Supplementary Material

Figure S1 Supplemental Figure 1. Effect of XO and Febuxostat (FX) on 2FA toxicity at 48 hours in two MTAP deleted pancreatic carcinoma cell lines.

Figure S2 Supplemental Figure 2. Effect of FX on cell growth after 48 hours.

Figure S3 Supplemental Figure 3. Weight loss and toxicity of HT1080 SCID mice.

Figure S4 Supplemental Figure 4. Analysis of MiaPaCa-2 SCID experiment.

Figure S5 Supplementary Figure 5. Analysis of MiaPaCa-2 nude mice experiment.

Supplementary Video 1 Supplementary Video 1

## Data Availability

The data generated in this study are available upon request from the corresponding author.

## References

[1] Samaras P , Schmidt T, Frejno M, Gessulat S, Reinecke M, Jarzab A, . ProteomicsDB: a multi-omics and multi-organism resource for life science research. Nucleic Acids Res 2020;48:D1153–63. 10.1093/nar/gkz974. 31665479 PMC7145565

[2] Carrera CJ , Eddy RL, Shows TB, Carson DA. Assignment of the gene for methylthioadenosine phosphorylase to human chromosome 9 by mouse-human somatic cell hybridization. Proc Natl Acad Sci U S A 1984;81:2665–8. 10.1073/pnas.81.9.2665. 6425836 PMC345130

[3] Olopade OI , Bohlander SK, Pomykala H, Maltepe E, Van Melle E, Le Beau MM, . Mapping of the shortest region of overlap of deletions of the short arm of chromosome 9 associated with human neoplasia. Genomics 1992;14:437–43. 10.1016/s0888-7543(05)80238-1. 1385305

[4] ICGC/TCGA Pan-Cancer Analysis of Whole Genomes Consortium . Pan-cancer analysis of whole genomes. Nature 2020;578:82–93. 10.1038/s41586-020-1969-6. 32025007 PMC7025898

[5] Clouser MC , Suh M, Movva N, Hildebrand JS, Pastula ST, Schoehl M, . A systematic literature review of MTAP deletions in solid and hematologic cancers. Cancer Treat Res Commun 2025;44:100966. 10.1016/j.ctarc.2025.100966. 40729867

[6] Fan N , Zhang Y, Zou S. Methylthioadenosine phosphorylase deficiency in tumors: a compelling therapeutic target. Front Cell Dev Biol 2023;11:1173356. 10.3389/fcell.2023.1173356. 37091983 PMC10113547

[7] Tang B , Lee HO, An SS, Cai KQ, Kruger WD. Specific targeting of MTAP-deleted tumors with a combination of 2′-fluoroadenine and 5′-methylthioadenosine. Cancer Res 2018;78:4386–95. 10.1158/0008-5472.CAN-18-0814. 29844120 PMC6072572

[8] Parker WB , Allan PW, Shaddix SC, Rose LM, Speegle HF, Gillespie GY, . Metabolism and metabolic actions of 6-methylpurine and 2-fluoroadenine in human cells. Biochem Pharmacol 1998;55:1673–81. 10.1016/s0006-2952(98)00034-3. 9634004

[9] Tang B , Testa JR, Kruger WD. Increasing the therapeutic index of 5-fluorouracil and 6-thioguanine by targeting loss of MTAP in tumor cells. Cancer Biol Ther 2012;13:1082–90. 10.4161/cbt.21115. 22825330 PMC3461815

[10] Gabitova-Cornell L , Surumbayeva A, Peri S, Franco-Barraza J, Restifo D, Weitz N, . Cholesterol pathway inhibition induces TGF-beta signaling to promote basal differentiation in pancreatic cancer. Cancer Cell 2020;38:567–83.e11. 10.1016/j.ccell.2020.08.015. 32976774 PMC7572882

[11] Campbell PM , Groehler AL, Lee KM, Ouellette MM, Khazak V, Der CJ. K-Ras promotes growth transformation and invasion of immortalized human pancreatic cells by Raf and phosphatidylinositol 3-kinase signaling. Cancer Res 2007;67:2098–106. 10.1158/0008-5472.CAN-06-3752. 17332339

[12] Rafehi H , Orlowski C, Georgiadis GT, Ververis K, El-Osta A, Karagiannis TC. Clonogenic assay: adherent cells. J Vis Exp 2011:2573. 10.3791/2573. 21445039 PMC3197314

[13] Jensen MM , Jørgensen JT, Binderup T, Kjaer A. Tumor volume in subcutaneous mouse xenografts measured by microCT is more accurate and reproducible than determined by 18F-FDG-microPET or external caliper. BMC Med Imaging 2008;8:16. 10.1186/1471-2342-8-16. 18925932 PMC2575188

[14] Zhang F , Wang Y. The efficacy and safety of different doses of febuxostat and allopurinol: a meta-analysis. Jt Dis Relat Surg 2025;36:562–76. 10.52312/jdrs.2025.1976. 40783988 PMC12456345

[15] Yu H , Chen X, Guo X, Chen D, Jiang L, Qi Y, . The clinical value of serum xanthine oxidase levels in patients with acute ischemic stroke. Redox Biol 2023;60:102623. 10.1016/j.redox.2023.102623. 36739755 PMC9932569

[16] Ghandi M , Huang FW, Jané-Valbuena J, Kryukov GV, Lo CC, McDonald ER 3rd, . Next-generation characterization of the Cancer Cell Line Encyclopedia. Nature 2019;569:503–8. 10.1038/s41586-019-1186-3. 31068700 PMC6697103

[17] Wyngaarden JB , Dunn JT. 8-Hydroxyadenine as the intermediate in the oxidation of adenine to 2, 8-dihydroxyadenine by xanthine oxidase. Arch Biochem Biophys 1957;70:150–6. 10.1016/0003-9861(57)90088-7. 13445250

[18] Tai LA , Hwang KC. Regulation of xanthine oxidase activity by substrates at active sites via cooperative interactions between catalytic subunits: implication to drug pharmacokinetics. Curr Med Chem 2011;18:69–78. 10.2174/092986711793979760. 21110814

[19] Simile MM , Banni S, Angioni E, Carta G, De Miglio MR, Muroni MR, . 5′-Methylthioadenosine administration prevents lipid peroxidation and fibrogenesis induced in rat liver by carbon-tetrachloride intoxication. J Hepatol 2001;34:386–94. 10.1016/s0168-8278(00)00078-7. 11322199

[20] Moreno B , Lopez I, Fernández-Díez B, Gottlieb M, Matute C, Sánchez-Gómez MV, . Differential neuroprotective effects of 5′-deoxy-5′-methylthioadenosine. PLoS One 2014;9:e90671. 10.1371/journal.pone.0090671. 24599318 PMC3944389

[21] Kataoka H , Yang K, Rock KL. The xanthine oxidase inhibitor Febuxostat reduces tissue uric acid content and inhibits injury-induced inflammation in the liver and lung. Eur J Pharmacol 2015;746:174–9. 10.1016/j.ejphar.2014.11.013. 25449036 PMC4281294

[22] Wei X , Kane WJ, Adair SJ, Nagdas S, Liu D, Bauer TW. PRMT5 identified as a viable target for combination therapy in preclinical models of pancreatic cancer. Biomolecules 2025;15:948. 10.3390/biom15070948. 40723820 PMC12292163

